# The Use of Social Media by Female Physicians in an International Setting: A Mixed Methods Study of a Group WhatsApp Chat

**DOI:** 10.1089/whr.2019.0015

**Published:** 2020-02-17

**Authors:** Halah Ibrahim, Pascale Anglade, Sawsan Abdel-Razig

**Affiliations:** ^1^ Department of Medicine, Sheikh Khalifa Medical City, Abu Dhabi, United Arab Emirates.; ^2^ Digestive Disease Institute, Cleveland Clinic Abu Dhabi, Abu Dhabi, United Arab Emirates.; ^3^ Department of Medicine, Lerner College of Medicine, Cleveland Clinic Foundation, Cleveland, Ohio.; ^4^ Department of Medicine, Langone School of Medicine, New York University, New York, New York.

**Keywords:** gender equity, social media, WhatsApp, women's group

## Abstract

***Background:*** The past decade has witnessed an increase in informal and bottom up driven “she-for-she” efforts, often using social media, to promote the advancement of women in medicine. Yet, this area of research is nascent with limited information on the use of social media platforms by female physicians, especially in the international medical arena. The purpose of this study was to investigate the use of a social media platform by a diverse group of female physicians in an international setting.

***Materials and Methods:*** The study used a mixed methods approach, including quantitative descriptive statistics and qualitative thematic analysis of the content of posts of a women physicians WhatsApp group during a 1-year time period (June 1, 2018–May 31, 2019).

***Results:*** The group consisted of 122 members with 4897 posts during the 1-year time period. Nine themes were identified including requests for medical information, logistics, personal recommendations, promotion, celebration, community engagement, education, women's empowerment, and employment inquiries. Engagement was high with 72% of members posting during the last 30 days of analysis and 92% of questions posted receiving a response, often within minutes. There were no instances of unprofessional social media behavior.

***Conclusions:*** The social media platform was effective in enabling female physicians to expand networks, exchange ideas, share scientific information, celebrate accomplishments, and provide support to colleagues. Creating a social media forum for women physicians may be an effective tool to foster a network of support and community.

## Introduction

Health care is a gendered field. The literature on gender inequity in medicine is large and robust, covering all specialties and spanning several decades.^1^ Studies document a lack of progress in closing the gender gap in wages, publication rates, recognition, promotion, and leadership.^2,3^ There is an ever-growing body of evidence that these inequities are not a pipeline issue, but rather due to a complex interplay of deeply entrenched institutional and societal biases against female physicians.^4^ These enduring gender-based inequities have spurred numerous initiatives to improve resources and work environments for women in health care. Yet, a recent survey found that 40% of respondents reported that their institutions lacked any programs to support the career advancement of women physicians.^5^ Formalized programs, when present, focus on individual interventions, such as mentoring, and have had mixed results.^5^ In recent years, there has been an increase in informal and bottom up driven “she-for-she” efforts, often using social media platforms, to promote the advancement of women in medicine.^6^

Social media can facilitate the breakdown of gender barriers in health care and empower female physicians to express and build important relationships.^6^ Virtual platforms are powerful tools to unite and maintain communities of women to advocate for greater representation and participation in the medical workforce. Studies have shown the role of Twitter, for example, in promoting women physician's needs and expanding dissemination of research by female scientists.^7^ A study of a closed Facebook group for the Hematology/Oncology Women Physician Group found that the platform was beneficial for both professional medical concerns and for social support.^8^ From a feminist theory lens, social media platforms may serve as important tools to promote inclusiveness and respect. This area of research, however, is nascent and there is limited information on the use and impact of many social media platforms on the lives and careers of female physicians, especially in the international medical arena.

WONDER Women Physicians group was established in 2015 to provide community and support among female physicians living and working internationally in the Middle East. Although the literature on gender and the medical workforce in the region is limited, existing studies suggest that women physicians face both explicit discrimination and implicit biases that contribute to the underrepresentation of women in health care leadership roles.^9^ Over the years, the women's group membership expanded through referrals from personal networks of members, resulting in a multispecialty network of female physicians. Meetings were held quarterly to allow for traditional networking and often included educational events.^10^ In February 2018, a closed group messaging application was added as an adjunct to face-to-face meetings, with the goal of expanding membership and providing a forum for communication of group events. The WhatsApp Messenger^11^ was selected because of its widespread use and ability to send free text, audio, and video to individual contacts or instantaneously to all members within a group. Increased cyber security from end-to-end encryption ensured that messages were securely transmitted. The use of WhatsApp by physicians has been highlighted in recent articles describing the advantages for collaboration across institutions to improve patient care^12^ and for individual physician education and professional development.^13^

The authors hypothesized that the WhatsApp group would promote camaraderie and emphasize shared experiences among members, thereby, creating a supportive online community for female physicians. The primary purpose of this study was to analyze the content of the social media interactions of members of this women physicians group based in an international setting. Additional aims included examination of levels of engagement and professionalism in these interactions.

## Materials and Methods

This study used a mixed methods approach, including quantitative descriptive statistics and qualitative thematic analysis. Since the data is retrospective, deidentified, and does not involve any patient information, the study was considered exempt from ethical approval by the Cleveland Clinic Abu Dhabi Institutional Review Board. Before data extraction and analysis, group members were informed of the retrospective study *via* a WhatsApp message and encouraged to privately message the authors (H.I. and P.A.) with concerns or whether they wanted their posts excluded from analysis. There were no objections from group members. Data were exported from the WhatsApp Messenger group to Microsoft Office Excel 2013 by one of the study authors (H.I.) who removed identifying information and assigned each member a unique numeric identifier. These data were then shared with the remaining authors (P.A. and S.A.R.). Posts from June 1, 2018, through May 31, 2019, were included in the analysis. Posts that contained only emojis, without text, graphics, or video, were excluded. Retrieved information from posts included content, date, and time of posts.

Descriptive quantitative analysis using Microsoft Excel Version 13 were used to analyze frequency and timing of posts per member and in aggregate.

Engagement was analyzed in terms of responsiveness to queries and, the proportion of members posting in the last 30 days and 7 days of analysis. Previous studies of social media engagement have used similar time frames.^8,13^ Responsiveness was determined by time elapsed between an original query post and the corresponding first reply. Minimum and maximum response time throughout the year was determined by sampling one question/answer pair per week (first question of each Monday over the 52-week period).

Two physician reviewers (H.I. and P.A.) performed an independent thematic content analysis of all WhatsApp posts. Each reviewer produced a list of common themes identified and listed messages that may qualify as unprofessional social media behavior (SMB). Unprofessional SMB was defined as posts (messages or images) that breached patient confidentiality, included profanity or discriminatory speech, or disparaged other doctors or the profession of medicine. At the end of this initial process, the reviewers convened and discussed the characteristics of the themes and created subthemes. They also compared findings of posts identified as unprofessional. Any disagreements were resolved by consensus. The two primary reviewers then independently reviewed and coded all posts according to the predetermined themes. All disagreements were resolved by consensus. Remaining discordance between the two primary reviewers was brought to the third physician reviewer (S.A.R.) and discussed until consensus was achieved.

## Results

### Quantitative results

From June 1, 2018, to May 30, 2019, 77 new members joined the closed WhatsApp chat group, whereas 6 women physicians exited the group, resulting in a total of 122 members. There were 4897 posts during the 1-year time period, of which 379 posts contained only emojis, without text or video, and were excluded from the qualitative analysis, resulting in 4518 posts for analysis. Eighty-eight unique members (72% of members) posted during the last 30 days of analysis and 32 members (26%) contributed within the last 7 days of analysis. Of the sampled questions, 92% received a response. Mean response time was 13 minutes and 52 seconds (standard deviation 19 minutes 58 seconds), with 52% of responses received in <5 minutes and 35% of responses in <2 minutes. Minimum and maximum response times were 16 seconds and 1 hour, 32 minutes, 5 seconds, respectively.

### Qualitative results

Nine themes were identified with frequency of each theme described in Table 1. The most frequent posts 36.8% (*n* = 1663) were requests for medical information, with the vast majority being physician referral requests. At 1.7% (*n* = 77), employment queries comprised the lowest number of posts.

**Table 1.** Nine Themes Derived from Qualitative Analysis of WhatsApp Group Chat

**Theme**	**Total no. of posts (%)**	**Description**	**Examples**
Requests for medical information	1663 (36.8)	Physician referral requests and medical or expert opinion questions posed to the group. None of the posts included patient identifying information	“Can the dermatologists in the group comment on this [photo] please? Does it look infected or like psoriasis?” “Good morning team wonder, am just wondering if we have any psychiatrists on the group or if anyone knows or works with someone they can put me in touch with? Many thanks”
Logistics	625 (13.8)	Administrator posts to announce group activities and events	“REMINDER: WONDER walk/run on is TOMORROW Wednesday! Meeting point: in front of the cafe (for easy parking) at 630pm with a dinner afterwards (740pm)”
Personal recommendations	502 (11.1)	Solicited and unsolicited recommendations for nonmedical services	“Quick ladies: good place for noodles? Nothing too fancy. My kid is requesting noodles for his bday and I have no clue where to bring him. Reasonable Chinese food somewhere??”
Promotion	483 (10.7)	Messages of self-promotion, recognition, and promotion of other female physicians, or promotion of the group as a whole	“I attended one of your lectures at the primary care conference last year. I think you're a brilliant speaker!” “I love this group for we are true women who stand by each other in spite not knowing each other well…” “…Haven't met most of you personally but do feel a strong kinship. Thanks for making our lives a lil’ bit brighter than before”
Celebration	384 (8.5)	Secular and religious holiday wishes, personal and professional milestones, and celebrations	“Happy Easter sisters. Happy Palm Sunday for those celebrating according to the Julian calendar” “And a very Happy Holi …May all of your lives be filled with colors of happiness, peace and love”
Community engagement	368 (8.1)	Nonmedical related, general posts, quotes, memes, videos, or articles of interest	“What colors are these sneakers? Right and left brain dominant: if you're right brain is dominant, you will see combination of pink and white color, and if your left brain is dominant, you will see it in gray and green color. Try with your loves ones and friends, very interesting”
Education	210 (4.6)	Articles of interest to the general medical community, websites, or brochures of local and regional medical conferences and seminars	“Beautiful article if you have 5 min to spare. We are torn between rules set by ourselves, by our institutions, lawyers, by our desire to protect ourselves, and by our instinct to bond and connect with another human being”
Women's empowerment	206 (4.6)	Articles regarding women in medicine, inspirational quotes, and images relating to women's empowerment	“Wishing all you talented WONDER docs an inspiring Women's Day. We juggle so much and work so hard and yet often wonder if we do enough. My wish and hope for the year ahead is that WONDER remains a place we can turn to ask questions, to be listened to, to be supported and to be reminded that you are more than enough. Let's give ourselves and each other grace as our Women's Day present”
Employment inquiries	77 (1.7)	Employment opportunity posts or networking posts related to searches for new employment	“We are looking for an operations manager for our outpatient services at [name removed] medical center. …Does anyone know anyone that would be interested? If so please let me know:) and thank you!:)” Dear all, I wonder if any of the facilities that you work in is looking to hire a consultant family medicine from the UK. I know a lovely friend who is a qualified GP… looking to move. I would appreciate your help. Thanks in advance

With regard to SMB, one of the primary reviewers had initially identified one potential breech of patient confidentiality; however, upon joint review, both reviewers noted that the poster had indicated receipt of patient consent to share identifying information. Therefore, this instance of potential breech of confidentiality was reconciled by consensus as acceptable SMB. Ultimately, no unprofessional SMB was identified.

## Discussion

This first study of the content of a social media chat group in an international women physicians group has revealed many important and encouraging insights. It is interesting that, by far, the most common topic of discussion was related to members' professional roles as physicians, as 37% of posts involved physician referral queries and requests for expertise sharing. The least common theme identified related to job inquiries (representing only 1.7% of posts). This was somewhat consistent with previous studies, which demonstrate gender differences in the utility of networking behavior toward career outcomes.^14^ It is also possible that members did not identify this social media platform as a potential venue for job opportunities or may be a reflection of the employment status of members.

Studies have shown that women physicians are offered fewer speakership invitations and receive less recognition for their accomplishments.^15–17^ As such, social media dissemination may help group members to build their professional reputations and portfolios. We are encouraged that group members chose to use this platform to help promote each other's work and achievements and disseminate presentations and research conducted by their female colleagues. A notable use of the platform is described by the “community building” theme identified in 8.1% of posts (*n* = 368 posts). This finding supports the notion that social media can, indeed, be a powerful tool to build an online community. In our study, the virtual platform transcended geographic barriers and hierarchal structures and enabled our female physicians to expand networks, exchange ideas, and foster connectedness. This is consistent with a recent study of junior doctors in the United Kingdom that found that WhatsApp helped to break down traditional hierarchies, improved communication, and fostered team cohesion, with a third of their messages consisting of advice or support.^18^

The WhatsApp platform demonstrated a high degree of member engagement (more than two-thirds of members posting within 30 days), with high response rates (92%) to questions posed by group members, and rapid responses to questions posed (occurring within minutes). We believe this likely represents a high degree of familiarity with this type social media platform, a significant sense of community and shared experience, and a general interest in topics discussed. It is likely that even those who do not actively post can also reap the benefits of the online community through passive observation and the ability to access content shared. The limited number of individuals who have left the group chat supports this belief. Furthermore, we are aware that some of these virtual connections have turned into live friendships, with many members posting pictures from social gatherings and convening in person at medical conferences to support other members' professional development.

Despite previous studies demonstrating a significant potential for unprofessional SMB, including patient privacy violations, profanity or discriminatory language, and posting sexually explicit material,^19–21^ review of this chat did not reveal any instances of unprofessional posts by our group members. In addition, although no “ground rules” were set at the initiation of the WhatsApp group, we believe that there was a grassroots effort by the participants to create a safe productive online space, emphasizing approaches rooted in feminism, including collaboration, reciprocity, community building, and respect.^22^ True to a feminist social media space, online interactions were productive and promoted solidarity and equality.

Limitations of this study include that it involves a single international woman physicians group, which may not be generalizable to other female physicians. Also, only group chat messages were analyzed; individual conversations between group members were not included. Furthermore, analysis did not measure the impact of either active or passive participation in the WhatsApp group chat on group members. It is our hope that the high level of engagement correlates with feelings of belonging and support. Surveys or interviews with group members can help determine whether the social media platform has fostered community or added value. Additional studies are needed to determine whether membership in the online women physicians group has had any impact on career satisfaction, decreased burnout, or fostered resilience in individual members. More work also needs to be done to assess whether online women's groups impact career development or contribute to the advancement of women in medicine.

## Conclusions

Closing the gender gap in medicine will require a multifaceted approach. Social media is becoming an increasingly important part of this approach. Our experience has shown that creating a social media forum for women physicians can foster a social network of support and community. Our hope is that formation of chat groups, and other social media platforms, will provide safe spaces for female physicians and, ultimately, contribute to improving the representation and participation of women in medicine.

## Abbreviation Used

SMB: social media behavior

## References

[1] El Arnaout N, Chehab RF, Rafii B, Alameddine M Gender equity in planning, development and management of human resources for health: A scoping review. Hum Resour Health 2019;17:52 31296235 10.1186/s12960-019-0391-3 PMC6625080

[2] Jena AB, Olenski AR, Blumenthal DM Sex differences in physician salary in US public medical schools. JAMA Intern Med 2016;176:1294–1304 27400435 10.1001/jamainternmed.2016.3284 PMC5558151

[3] Elinas EH, Kaljo K, Patitucci TN, Novalija J, Byars-Winston A, Fouad NA No room to “lean in”: A qualitative study on gendered barriers to promotion and leadership. J Womens Health (Larchmt) 2019;28:393–402 30481114 10.1089/jwh.2018.7252

[4] Silver JK, Rowe M, Sinha MS, et al. Micro-inequities in medicine. PM R 2018;10:1106–1114 30366648 10.1016/j.pmrj.2018.08.382

[5] Carr PL, Gunn C, Raj A, Kaplan S, Freund KM Recruitment, promotion, and retention of women in academic medicine: How institutions are addressing gender disparities. Womens Health Issues 2017;27:374–381 28063849 10.1016/j.whi.2016.11.003 PMC5435548

[6] Shillcutt SK, Silver JK Social media and advancement of women physicians. N Engl J Med 2018;378:2342–2345 29897857 10.1056/NEJMms1801980

[7] Cawcutt KA, Erdahl LM, Englander MJ, et al. Use of a coordinated social media strategy to improve dissemination of research and collect solutions related to workforce gender equity. J Womens Health (Larchmt) 2019;28:849–862 30998087 10.1089/jwh.2018.7515

[8] Graff SL, Close J, Cole S, Matt-Amaral L, Beg R, Markham MJ Impact of closed Facebook group participation on female hematology/oncology physicians. J Oncol Pract 2018;14:e758–e769 30537459 10.1200/JOP.18.00448

[9] Hannawi S, Al Salmi I Time to address gender inequalities against female physicians. Int J Health Plann Manage 2018;33:532–541 29124785 10.1002/hpm.2476

[10] Ibrahim H, Stadler D, Archuleta S, Anglade P, Cofrancesco J Jr. Twelve tips to developing and running a successful women's group in international academic medicine. Med Teach 2019;41:1239–1244 30428757 10.1080/0142159X.2018.1521954

[11] WhatsApp. Available at: https://www.whatsapp.com Accessed 1110, 2019

[12] Othman M, Menon V Developing a nationwide spine care referral programme on the WhatsApp messenger platform: The Oman experiment. Int J Med Inform 2019;126:82–85 31029267 10.1016/j.ijmedinf.2019.03.019

[13] Raiman L, Antbring R, Mahmood A WhatsApp messenger as a tool to supplement medical education for medical students on clinical attachment. BMC Med Educ 2017;17:7 28061777 10.1186/s12909-017-0855-x PMC5219809

[14] Forret ML, Dougherty TW Networking behaviors and career outcomes: Differences for men and women? J Organ Behav 2004;25:419–437

[15] Lincoln AE, Pincus S, Koster JB, Leboy PS The Matilda effect in science: Awards and prizes in the US, 1990s and 2000s. Soc Stud Sci 2012;42:307–320 22849001 10.1177/0306312711435830

[16] Silver JK, Slocum CS, Bank AM, et al. Where are the women? The underrepresentation of women physicians among recognition award recipients from medical specialty societies. PM R 2017;9:804–815 28606837 10.1016/j.pmrj.2017.06.001

[17] Ibrahim H, Abdel-Razig S, Stadler DJ, Cofrancesco J Jr, Archuleta S Assessment of gender equity among invited speakers and award recipients at US annual medical education conferences. JAMA Netw Open 2019;2:e1916222 31774518 10.1001/jamanetworkopen.2019.16222 PMC6902833

[18] Gould G, Nilforooshan R WhatsApp Doc? BMJ Innov 2016;2:109–110 10.1136/bmjinnov-2016-000116 PMC4975831 27547446

[19] Chretien KC, Azar J, Kind T Research letter: Physicians on Twitter. JAMA 2011;305:566–568 21304081 10.1001/jama.2011.68

[20] Brynolf A, Johansson S, Appelgren E, Lynoe N, Edstedt Bonamy AK Virtual colleagues, virtually colleagues—physicians' use of Twitter: A population-based observational study. BMJ Open 2013;3:e002988 10.1136/bmjopen-2013-002988 PMC3731708 23883885

[21] Soares W, Shenvi C, Waller N, Johnson R, Hodgson CS Perceptions of unprofessional social media behavior among emergency medicine physicians. J Grad Med Educ 2017;9:85–89 28261400 10.4300/JGME-D-16-00203.1 PMC5319635

[22] Knoll MA, Jagsi R Social media and gender equity in Oncology. JAMA Oncol 2019;5:15–16 30452515 10.1001/jamaoncol.2018.4647

